# Current antibiotic resistance patterns of rare uropathogens: survey from Central European Urology Department 2011–2019

**DOI:** 10.1186/s12894-021-00821-8

**Published:** 2021-04-13

**Authors:** Jan Hrbacek, Pavel Cermak, Roman Zachoval

**Affiliations:** 1 Department of Urology, 3rd Faculty of Medicine, Charles University and Thomayer University Hospital, Videnska 800, 140 59 Prague, Czech Republic; 2 Department of Clinical Microbiology, Thomayer University Hospital, Videnska 800, Prague, 14059 Czech Republic

**Keywords:** Urinary tract infections, Drug resistance, Bacterial, Anti-infective agents, Urinary, Acinetobacter, Citrobacter, Enterobacter, Morganella, Providencia, Serratia, Stenotrophomonas

## Abstract

**Background:**

While the resistance rates of commonly detected uropathogens are well described, those of less frequent Gram-negative uropathogenic bacteria have seldom been reported. The aim of this study was to examine the resistance rates of less frequent uropathogenic Gram-negatives in a population of patients treated in a Department of Urology of a tertiary referral centre in Central Europe over a period of 9 years.

**Methods:**

Data on all positive urine samples from urological in- and out-patients were extracted form the Department of Clinical Microbiology database from 2011 to 2019. Numbers of susceptible and resistant isolates per year were calculated for these uropathogens: *Acinetobacter* spp. (*n* = 74), *Citrobacter* spp. (*n* = 60), *Enterobacter* spp. (*n* = 250), *Morganella morganii* (*n* = 194), *Providencia* spp. (*n* = 53), *Serratia* spp. (*n* = 82) and *Stenotrophomonas maltophilia* (*n* = 27). Antimicrobial agents selected for the survey included: ampicillin, amoxicillin/clavulanic acid, piperacillin/tazobactam; cefuroxime, cefotaxime, ceftazidime and cefepime; ciprofloxacin and ofloxacin; gentamicin and amikacin; ertapenem, meropenem and imipenem; trimethoprim-sulfamethoxazole (co-trimoxazole), nitrofurantoin and colistin.

**Results:**

Penicillin derivatives have generally poor effect except piperacillin/tazobactam. Cefuroxime is not efficient unlike cefotaxime (except against *Acinetobacter* spp. and *S. maltophilia*). Susceptibility to fluoroquinolones is limited. Amikacin is somewhat more efficient than gentamicine but susceptibilities for both safely exceed 80%. Nitrofurantoin shows virtually no efficiency. Cotrimoxazole acts well against *Citrobacter* spp., *Serratia* spp. and it is the treatment of choice for *S. maltophilia* UTIs. Among carbapenems, ertapenem was less efficient than meropenem and imipenem except for *S. maltophilia* whose isolates were mostly not suceptible to any carbapenems.

**Conclusions:**

Uropathogenic microorganisms covered in this report are noteworthy for their frequently multi-drug resistant phenotypes. Knowledge of resistance patterns helps clinicians choose the right empirical antibiotic treatment when the taxonomical assignment of the isolate is known but sensitivity results are pending.

**Supplementary Information:**

The online version contains supplementary material available at 10.1186/s12894-021-00821-8.

## Background

With approximately 150–250 million cases occurring globally per year [1, 2], urinary tract infections (UTIs) represent some of the most common infectious diseases in humans. Gram-negative enteric bacteria such as *Escherichia coli*, *Klebsiella* spp. and *Proteus* spp. are their most common causative agents. UTIs occur more frequently among women than in men: 50% of females will experience at least one UTI in their lifetime [3]. Recurrences are common and besides associated morbidity, UTIs put pressure on health care systems, too. While the cost of treatment of a single UTI episode is insignificant, their frequent incidence means they consume a non-negligible part of the health care budget: in France alone, the expense for adult female UTIs amounted to 58 million euro in 2012 [4]. Complicated UTIs are even more costly: one episode of a complicated UTI costs between 4028 and 7740 euro [5] depending on the health care system.

Antimicrobial resistance (AMR) has become a major concern threathening many an advance of medicine in the twenty-first century. Uropathogenic microorganisms are no exception to this: rates of AMR among Enterobacteriaceae are increasing globally, albeit with temporal and spatial differences [6–15]. Antibiotic consumption is a primary driver for the spread of AMR as evidenced on local and country levels [16, 17]. For instance, southern European states exhibit higher AMR rates than northern ones. Of note, four top antibiotic prescribers in Europe (Greece, Cyprus, France and Italy) belong among south European countries [18].

International guidelines for UTI treatment are freely available; yet, poor antibiotic prescription remains common. Thirty percent of primary care antibiotic prescriptions were inadequate in a report from the United States [19]. Appropriate antibiotic treatment was reported in 68% of adult cystitis cases and only 46% of pyelonephritis cases in another survey [20].

One of the ways to improve the quality of antibiotic prescribing is antibiotic stewardship including the monitoring of local antimicrobial susceptibility patterns [6]. While AMR patterns of the commonest causative uropathogenic agents—*E. coli*, *Klebsiella* spp., *Proteus* spp., *Pseudomonas aeruginosa* etc. have been widely reported, less attention has been devoted to microbes at the bottom of the prevalence ladder. These microorganisms constitute only a small fraction of UTI etiological agents but frequently display a multi-drug resistant phenotype, presenting a therapeutic challenge [21, 22]. Furthermore, they often have the ability to survive in hostile environments such as dry surfaces, nutrient-poor aqueous solutions [23, 24] and require a particularly attentive nursing care.

The aim of this retrospective observational study was to report the AMR patterns for seven less frequent causative agents of UTIs in a Central European tertiary referral centre Department of Urology over a period of nine years.

## Methods

Similar to our previous related work [25], urinary cultures from the Department of Clinical Microbiology electronic database between January 2011 and December 2019 were searched for those caused by the following genera: *Acinetobacter*, *Citrobacter*, *Enterobacter*, *Morganella*, *Providencia*, *Serratia* and *Stenotrophomonas*. Urine cultures may have originated from spontaneously voided midstream samples, aseptic catheterisation during theatre procedures, indwelling catheters, suprapubic catheters, nephrostomy tubes and uretero-ileostomies. Because of the small prevalence of these uropathogens, inpatient and outpatient samples were included in the analysis. Duplicates were eliminated, allowing only one isolate of a given pathogen per patient per year. Prevalence of uropathogenic organisms and their antimicrobial susceptibility patterns (including trends where appropriate) were analysed.

### Culture methods and susceptibility testing

The method used for culture and susceptibility testing has been described elsewhere [25]. Briefly, uncentrifuged urine was inoculated with a 0.01 ml loop on blood and UriSelect chromogenic agar (Bio-Rad, Berkeley, CA, USA) in a semi-quantitative dilution method. Urine diluted in saline (1:10) was inoculated with a 0.01 mL and 0.001 mL loop on Columbia blood agar (Bio-Rad, Berkeley, CA, USA) and UriSelect chromogenic agar. Agar plates were incubated at 37 °C for 20–24 h. Microorganisms were identified by their phenotypical characteristic and using the semi-automatic system MIKROLATEST ID (Erba-Lachema, Brno, Czech Republic).

Antibiotic susceptibility testing was performed by the disc diffusion method on Mueller–Hinton agar (Bio-Rad, Berkeley, CA, USA) and by the MIC dilution method (TRIOS MIC, Prague, Czech Republic until 2017, then MICRO-LA-TEST ATB (MIC) Erba-Lachema, Brno, Czech Republic). EUCAST MIC breakpoint tables were used. Intermediate results were excluded from this analysis. The cut-off used for significant bacterial presence was 10^5^ colony-forming units/mL (10^3^ for aseptically catheterised urine specimens).

This survey covers the following antimicrobial agents: ampicillin, amoxicillin/clavulanate and piperacillin/tazobactam; cefuroxime, cefotaxime, ceftazidime and cefepime; ciprofloxacin and ofloxacin; gentamicin and amikacin; ertapenem, meropenem and imipenem; trimethoprim-sulfamethoxazole (co-trimoxazole), nitrofurantoin and colistin.

Cochrane-Armitage test was used to assess statistical significance of trends. Statistical analyses were performed in XLSTAT 2020.1.3 (Addinsoft, New York, USA). Alpha level of 0.05 was considered significant.

## Results

From a total of 15,909 positive urine cultures (duplicates excluded) between 1 January 2011 and 31 December 2019, the pathogens surveyed in the present study were detected in 740 samples (4.65%, range 3.33–6.03% of all positive urine cultures per year, no evidence for trend in incidence rate [p = 0.23]) from 607 patients. Of these, 466 (76.8%) were males and 141 (23.2%) were females. Mean age (interquartile range) was 70.3 (64–77) and 69.2 (59–76) for men and women, respectively. Two-thirds of urine samples (n = 482) originated from out-patients and 258 from in-patients (see Additional file 2: Table S7 for details). *Enterobacter* spp. was the most prevalent uropathogen during the study period (n = 250, 33.8%) followed by *Morganella morganii* (n = 194, 26.2%). The isolates identified included *Acinetobacter* spp. (n = 74), *Citrobacter* spp. (n = 60, including *C. braakii* (n = 1), *C. freundii* (n = 2), *C. koseri* (n = 23)), *Enterobacter* spp. (n = 250, including *E. aerogenes* (n = 3), *E. cloacae* (n = 12)), *Morganella morganii* (n = 194), *Providencia* spp. (n = 53, including *P. alcalifaciens* (n = 2), *P. rettgeri* (n = 11), *P. stuartii* (n = 32)), *Serratia* spp. (n = 82, including *S. ficaria* (n = 4), *S. marcescens* (n = 10), *S. odorifera* (n = 1)) and *Stenotrophomonas maltophilia* (n = 27).

**Fig. 1:**
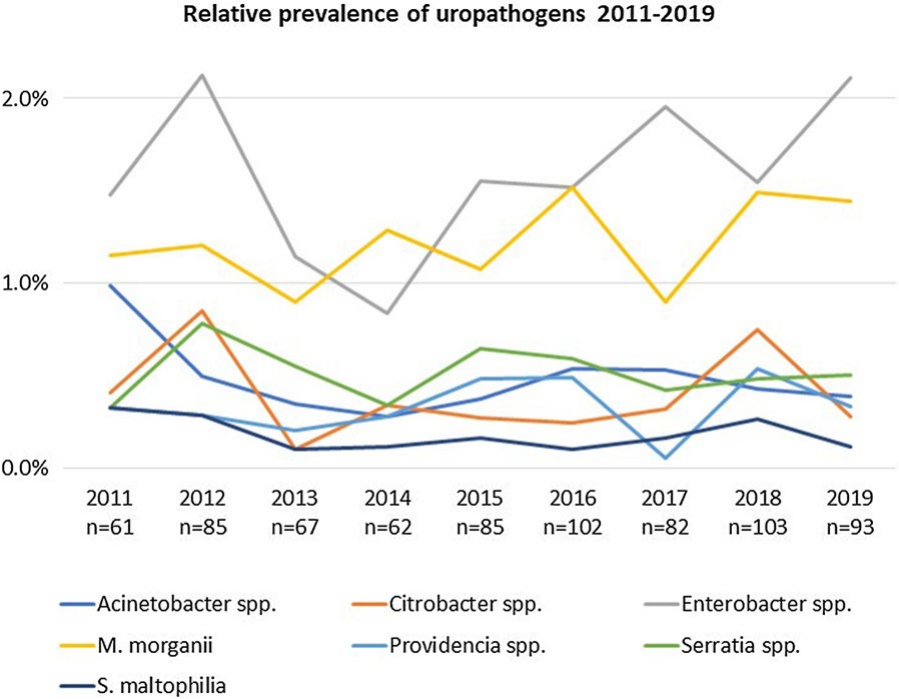
Prevalence of uropathogens during the study period (relative proportions of all positive urine samples)

There was no trend in the yearly prevalence of individual uropathogens (Fig. 1). Trends in resistance rates were computed for *Enterobacter* spp. and *M. morganii* where the number of isolates was sufficient to justify such analysis (Additional file 1: Table S1–S6).

### Antimicrobial resistance

#### Penicillin derivatives

Cumulative resistance rates calculated for the entire study period exceeded 80% for ampicillin and amoxicillin/clavulanic acid for all uropathogens. Piperacillin/tazobactam resistance did not exceed 10% in *Citrobacter* spp. and *Providentia* spp.; it was 16.7%, 15.4% and 13.6% for *Acinetobacter* spp., *Serratia* spp. and *M. morganii*, respectively. Table 1 summarizes resistance rates for the entire study period. Additional file 1: Table S1 gives details on the yearly number of isolates over the study period and their resistance rates to penicillins.

**Table 1:**
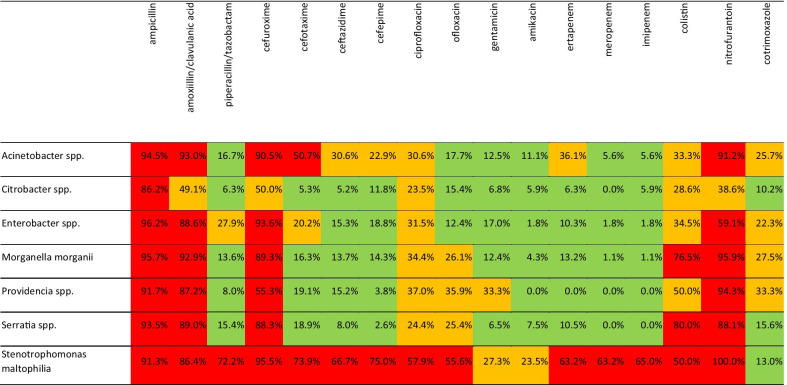
Cumulative resistance rates (2011–2019) of seven uropathogens to different antimicrobial agents

Numbers refer to the percentage of resistant isolates of a pathogen to a particular antibiotic during the study period. Colour coding: green = resistance rate < 20%; yellow = resistance rate 20–50%; red = resistance rate > 50%

#### Cephalosporines

All uropathogens were resistant (> 50%) to second-generation cephalosporine, cefuroxime. *Citrobacter* spp. susceptibility to 3rd generation cephalosporines, cefotaxime and ceftazidime approximated 5%. *Serratia* spp. was reasonably susceptible to ceftazidime and cefepime. *Providentia* spp. retained good susceptibility to cefepime. See Additional file 1: Table S2, for details on cephalosporine resistance rates.

#### Fluoroquinolones

Resistance rates of all uropathogens to ciprofloxacin exceeded 20% and approximated 60% in the case of *S. maltophilia*. Resistance rates to ofloxacin were somewhat lower but never less than 15%. Additional file 1: Table S3 presents details on fluoroquinolone resistance rates.

#### Aminoglycosides

*Citrobacter* spp. and *Serratia* spp. resistance to gentamicin was just above 6%. Resistance rates of the other uropathogens to gentamicin varied between 12.4% and 33.3%. All but *Acinetobacter* spp. and *S. maltophilia* retained good susceptibility to amikacin (see Additional file 1: Table S4 for details).

#### Carbapenems

While *Citrobacter* spp. and *Providentia* spp. retained good suceptibility (< 10%) to all carbapenems, resistance rates to ertapenem exceeded 10% in all other bacteria examined. *S. maltophilia* resistance rate to all three carbapenems was above 60%; Additional file 1: Table S5 gives a detailed overview.

#### Colistin, nitrofurantoin and cotrimoxazole

Colistin had poor effect on the pathogens surveyed in this report and their resistance rates exceeded 30% except *Citrobacter* spp. (28.6%). Resistance rate to nitrofurantoin approached 100% for all but *Citrobacter* spp. (38.6%). Cotrimoxazole resistance rates fluctuated between 10 and 30%. Cotrimoxazole was, however, the antibiotic *S. maltophilia* had the highest susceptibility to (Additional file 1: Table S6).

## Discussion

The uropathogens described in this report taken all together represent 5% of all positive urinary cultures from our urology department during the nine-year study period. Their low incidence likely explains the paucity of data in the literature on their prevalence and resistance patterns compared to other Gram-negative causative agents of UTIs. Some authors report resistance patterns for groups of these bacteria only [1, 26]; others focus on a single species within a genus [27, 28]. This is where discrepancies between our and other authors’ resistance data may stem from, apart from true differences in antimicrobial resistance observed across the world [6–15]. General informaion on the microorganisms covered in this report that is relevant to the topic is summarised in Table 2.

**Table 2:** Summary of clinically relevant characteristics of the microorganisms covered in this work

*Acinetobacter* spp.	Originally presumed to be of little pathogenicity, *Acinetobacter* has emerged as a troublesome etiological agent of hospital-acquired infections worldwide. This strictly aerobic Gram-negative coccobacillus is able to accumulate diverse mechanisms of resistance leading to phenotypes resistant to most commercially available antibiotics. The mechanisms implicated in *Acintetobacter* spp. resistance include (1) decreased expression of bacterial porins, hindering the passage of beta-lactams into the periplasmic space (where they can attach to penicillin-binding proteins); (2) over-expression of bacterial efflux pumps, causing decreased concentration of beta-lactams (as well as quinolones and other antibiotics) in the periplasmic space; (3) mutations in *gyrA* and *parC* genes causing resistance to fluoroquinolones; (4) expression of aminoglycoside-modifying enzymes leading to resistance to this class of antibiotics; (5) chromosomally encoded inducible AmpC beta-lactamases conferring resistance to cephalosporins. Finally, *Acinetobacter* spp. acquired (plasmid-mediated) serine- and metallo-beta-lactamases confer resistance to carbapenems [23, 32].
	Nosocomial UTIs are less frequent (most common *Acinetobacter*-related infection being ventilator-associated pneumonia) and are commonly diagnosed in elderly patients in ICU’s, mostly men with indwelling catheters [23]. *Acinetobacter* survives for long periods on wet and dry surfaces and several studies have documented extensive contamination of the environment in the vicinity of colonised patients: bed linen, bed curtains, sink traps and hospital floor [23].
*Citrobacter* spp.	*Citrobacter*, *Enterobacter* and *Serratia* are facultative anaerobic non-spore forming Gram-negative rods sometimes referred to as “CES” group and described together, due to the traits they share: (1) their biochemical characteristics; (2) prevalence and resistance patterns (observation corroborated by our data); (3) various intrinsic and acquired resistance mechanisms; and (4) the fact of being frequently associated with complicated UTIs, disease recurrence and prolonged treatment. Because of their AmpC beta-lactamase production, some authors group them with *Pseudomonas* spp. and indole-positive *Proteus* spp. to complete so-called “SPICE” group [33].
	*Citrobacter* spp. is also one of the microorganisms implicated in the “purple urine bag syndrome”, a conspicuous phenomenon of dubious clinical significance [34].
*Enterobacter* spp.	The genus Enterobacter comprises of 22 species and is considered an opportunistic pathogen. While its pathogenicity and virulence remain rather unclear, its resistance mechanisms have been extensively studied. The production of beta-lactamase is a major mechanism of resistance to beta-lactams; *E. aerogenes* expresses AmpC beta-lactamase (cephalosporinase) that confers resistance to 1st generation cephalosporins but is inducible during treatment with a 3rd generation cephalosporin, leading again to resistance. Aminoglycoside-modifying enzymes are responsible for resistance to aminoglycosides and a mutation in one of their genetic determinants (*aac-6*′*-Ib*) leads to a fluoroquinolone-resistant phenotype. A change in porin expression in the presence of imipenem leads to a decreased penetration of beta-lactams into *E. aerogenes* and bacterial efflux pumps remove fluoroquinolones and tetracyclines from *Enterobacter* spp. isolates [35]. Enterobacter is an opportunistic nosocomial pathogen: in a report from Taiwan, *E. cloacae* hospital outbreak has been described, attributale to a contaminated ureteroscope [36].
*Serratia* spp.	Originally presumed a non-pathogenic microorganism and even used a tracer in medical experiments, *Serratia* has established itself as an accepted clinical pathogen with widely prevalent multi-antibiotic resistant strains [37]. In urology wards, nosocomial outbreaks of *S. liquefaciens* and *S. marcescens* have been described related to cystometry appliance and urine bottles, respectively [37, 38]. Indeed, urine-measurement containers, urinometers, urine-collecting basins and urinals as well as cystoscopy suite have been found to be reservoirs of *S. marcescens* [37].
*Morganella* spp. and *Providencia* spp.	*Morganella* and *Providencia* are sometimes (together with *Proteus*) grouped under the tribe *Proteae*. Originally not considered frank pathogens, they have emerged as importang causative agents of hospital-acquired infections in different organ systems [39]. Their common characteristics include (1) strong urease production; (2) frequent association with complicated UTIs, prolonged treatment and disease recurrence; (3) intrinsic resistance to nitrofurantoin and colistin (as confirmed by our data) and to tetracycline; (4) intrinsically decreased susceptibility to imipenem (not observed on our data); and (5) production of various beta-lactamases including AmpC [26].
	Providencia frequently colonizes indwelling catheters; in a case-series (n = 14) of *Providencia* bacteraemia, UTI was identified as the source in 36% of cases and 71% of patients had an indwelling urinary catheter [40].
	*M. morganii*, *P. stuartii* and *P. rettgeri* are among the causative microorganisms of the purple-urine bag syndrome (see above).
*S. maltophilia*	*S. maltophilia* intrinsic resistance mechanisms (low membrane permeability, chromosomally encoded multidrug resistance efflux pumps, beta-lactamases, antibiotic-modifying enzymes etc.) were suggested to have been acquired in non-human natural environments and not being due solely to clinical use of antibiotics. Its survival is facilitated by wet surfaces and aqueous solutions with minimal nutrients (e.g. drinking water, treated water, dialysate effluent) [24].

In the present study, *Acinetobacter* resistance rate exceeded 30% for most antibiotics including ertapenem. It retained > 90% susceptibility to meropenem and imipenem and its resistance rates to piperacillin/tazobactam, ofloxacin and both aminoglycosides were within the clinically useful range of < 20% (Table 1). Thus, our Acinetobacter isolates were much more susceptible to most antimicrobials (except colistin) in comparison to a Spanish study from 2018 [29].

There is a limited amount of data on antimicrobial susceptibility of *Citrobacter* in clinical urinary isolates; Jiménez-Guerra et al. report a 10-year resistance patterns in urine samples (n = 65) from a tertiary Spanish hospital. Their resistance rates oscillate in line with ours except our three-fold and 5.5-fold higher resistance rate to cefepime and nitrofurantoin, respectively [28]. Of note, solely *C. freundii* was included in the Spanish study which may account for this difference. Fajfr et al. report on *Citrobacter* spp. (n = 97) resistance rates for ampicillin, ciprofloxacin and cotrimoxazole similar to our data [27].

*Enterobacter*, the most frequent pathogen in our study, retained extremely good susceptibility (> 90%) to amikacin, meropenem and imipenem only; clinically useful susceptibility rates were seen for ceftazidime, cefepime, ofloxacin, aminoglycosides and ertapenem. Cefepime resistance has shown a decreasing trend but this may be related to small numbers of *Enterobacter* isolates in the first half of the study period (Additional file 1: Table S2). A study from Asia–Pacific region reported similar resistance rates for cefepime and ertapenem; other antibiotics had different resistance profiles [15]. In comparison to a geographically closer Jiménez-Guerra et al. report [28], our *Enterobacter* strains were less resistant to imipenem (1.8% vs. 8%) and more resistant to ciprofloxacin (31% vs. 19%). The inclusion of only a particular species (*E. cloacae*) in the Spanish study may account for these differences.

Our *Serratia* spp. isolates exhibited highest resistance rates to ampicillin, amoxicillin/clavulanic acid, 2nd generation cephalosporins, colistin and nitrofurantoin, in line with its characteristics described in the literature [1]. Gajdács and Urbán [1] report on the cumulative resistance rates (n = 1132 in- and out-patients combined) of CES pathogens (Citrobacter, Enterobacter, Serratia) which are very close to our population (n = 392 in- and out-patients combined): 28.8% versus 20.2%, 13.3% versus 17.0% and 19.1% versus 18.0%, for ciprofloxacin, gentamicin and cotrimoxazole, respectively.

*Providencia* spp. had been presumed to be more resistant to antimicrobial drugs than *Morganella* spp. [26], but neither our nor others’ data [26] support this general assumption. *Providencia* spp. retained very good susceptibility to piperacillin/tazobactam and cefepime and no resistant strains were isolated to amikacin and all carbapenems. No report on urinary isolates AMR rates for *Providencia* was found in the literature. For *M. morganii*, Jiménez-Guerra et al. report twice lower resistance rates to ciprofloxacin and cotrimoxazole and ten times higher resistance to imipenem (11% vs. 1.1%) [28] when compared to our data. *M. morganii* resistance rates for some of the relevant antibiotics (specifically cefotaxime, ceftazidime, amikacin and ertapenem) showed statistically significant increasing trends. Interestingly, resistance to cotrimoxazole was decreasing (Additional file 1: Table S2, S4–S6).

Although the majority of infections caused by *S. maltophilia* are respiratory tract infections [24, 30]—it is the least prevalent uropathogen in the present study—its significance as causative agent of UTIs should not be underestimated; not for its prevalence but more so for its far-reaching resistance. In our study, *S. maltophilia* resistance rate exceeded 50% for all antibiotics tested (including carbapenems); only aminoglycosides and cotrimoxazole showed more favourable resistance profiles. Its resistance to cotrimoxazole was in fact among the lowest in the present survey, despite a (statistically non-significant) increasing trend in our and others’ data [31].

The limitations of the present report that need to be acknowledged include (1) the combination of both in- and out-patients in the analysis; due to small numbers of isolates cultured from urinary samples, the authors did not find further division of the study population feasible (interested readers can find the division into in- and out-patients in Additional file 2: Table S7); (2) the impossibility to distinguish between community and hospital-acquired infections as the dates of urine cultures could not be linked to admission and discharge dates of each in-patient episode; (3) lack of identification of isolates down to species level. It might also be insightful to discriminate urine samples representing asymptomatic bacteriuria as opposed to a clinically symptomatic UTI but the nature of our retrospective data would not allow for this. Our report lacks information on fosfomycin due to the fact this agent has not been used in our health care system and therefore not tested in the microbiology laboratory.

To our knowledge, this report is one of the few in the literature systematically describing prevalence and resistance patterns of uropathogens covered herein. Similarity of our resistance data to those originating from not-so-distant regions (in instances where a direct comparison was possible) suggests that our data are applicable in a wider geographic area and not just in our own institution.

Monitoring of antibiotic resistance patterns is an important contribution to (1) providing a better service to patients by the likely best choice of antimicrobial empirical treatment; (2) economically rational allocation of resources in healthcare; and (3) the prevention of the global rapid spread of antibiotic resistance, a phenomenon that jeopardises many advances in medicine and surgery achieved over the last century. The knowledge of most efficiently acting antimicrobial agents (piperacilin/tazobactam, carbapenems) should however not lead to their indiscriminate use as empirical treatment—perhaps with the exception of the gravest clinical scenarios.

## Conclusions

The uropathogens described in this report are important microorganisms not for their prevalence but for their high resistance rates to a majority of commonly used antibiotics. Penicillin derivatives have generally poor effect except piperacillin/tazobactam. Cefuroxime is not efficient unlike cefotaxime, ceftazidime and cefepime (except against *Acinetobacter* spp. and *S. maltophilia*). Susceptibility to fluoroquinolones is limited. Amikacin is somewhat more efficient than gentamicine but susceptibilities for both safely exceed 80%. Nitrofurantoin shows virtually no efficiency. Cotrimoxazole acts well against *Citrobacter* spp., *Serratia* spp. and it is the treatment of choice for *S. maltophilia* UTIs. Among carbapenems, ertapenem was less efficient than meropenem and imipenem except for *S. maltophilia* whose isolates were mostly not suceptible to any carbapenems.

## Supplementary Information

**Additional file 1.**  **Tables S1-S6:** Yearly antimicrobial resistance rates of uropathogens to different antibiotics.

**Additional file 2.** **Table S7:** Yearly numbers of isolates for each uropathogen in the in-patient and out-patient population.

## Data Availability

The anonymised raw datasets used and/or analysed during the current study are available from the corresponding author on reasonable request.

## Abbreviations

AMR: Antimicrobial resistance
CES: Citrobacter, Enterobacter, Serratia
EUCAST: The European Committee on Antimicrobial Susceptibility Testing
ICU: Intensive care unit
MIC: Minimal inhibitory concentration
SPICE: Serratia, Pseudomonas, indole-positive Proteus, Citrobacter, Enterobacter
UTI: Urinary tract infection
